# Biotechnological Trends in Spider and Scorpion Antivenom Development

**DOI:** 10.3390/toxins8080226

**Published:** 2016-07-23

**Authors:** Andreas Hougaard Laustsen, Mireia Solà, Emma Christine Jappe, Saioa Oscoz, Line Præst Lauridsen, Mikael Engmark

**Affiliations:** 1 Department of Biotechnology and Biomedicine, Technical University of Denmark, DK-2800 Kgs. Lyngby, Denmark; mireia.sc9@gmail.com (M.S.); emma.cjappe@gmail.com (E.C.J.); saio_jere@hotmail.com (S.O.); turtle_line@hotmail.com (L.P.L.); miken@bio.dtu.dk (M.E.); 2 Department of Drug Design and Pharmacology, Faculty of Health and Medical Sciences, University of Copenhagen, DK-2100 Copenhagen East, Denmark; 3 Department of Bio and Health Informatics, Technical University of Denmark, DK-2800 Kgs. Lyngby, Denmark

**Keywords:** antivenom, spider venom, scorpion venom, antitoxin, venom neutralization, venomics, antibodies, antivenom design

## Abstract

Spiders and scorpions are notorious for their fearful dispositions and their ability to inject venom into prey and predators, causing symptoms such as necrosis, paralysis, and excruciating pain. Information on venom composition and the toxins present in these species is growing due to an interest in using bioactive toxins from spiders and scorpions for drug discovery purposes and for solving crystal structures of membrane-embedded receptors. Additionally, the identification and isolation of a myriad of spider and scorpion toxins has allowed research within next generation antivenoms to progress at an increasingly faster pace. In this review, the current knowledge of spider and scorpion venoms is presented, followed by a discussion of all published biotechnological efforts within development of spider and scorpion antitoxins based on small molecules, antibodies and fragments thereof, and next generation immunization strategies. The increasing number of discovery and development efforts within this field may point towards an upcoming transition from serum-based antivenoms towards therapeutic solutions based on modern biotechnology.

## 1. Introduction

Scorpion stings and spider bites are a major public health concern in developing parts of the world, yet envenomation from these creatures still remains a neglected tropical disease [1]. Scorpionism affects people in Northern Africa, the Middle East, Central and South America, and to some extent India [2,3]. In comparison, spider bites mainly affect people in the Americas, Australia, and Africa [4], although bites are also known to occur in Europe [5]. In the scorpion order, nearly 2000 species are recognized [3], which is significantly less than the 44,000 recognized spider species [6]. Fortunately, only a subset of these species are of medical relevance, with the members of the Buthidae family (including bark scorpions and fat-tailed scorpions), *Latrodectus* genus (widow spiders) and *Loxosceles* genus (recluse spiders) being the main species with venom that may cause harm to humans [6,7].

Scorpions are considered the second most dangerous venomous animals to humans (after snakes), and their stings mainly affect children and adolescents [6]. Effective treatment against envenomings from the most venomous scorpions and spiders consists of parental administration of animal-derived antisera by medically trained personnel. Currently, there are 19 antivenoms for human use and one antivenom for animal use on the market for scorpion stings, whereas only 10 antivenoms are used clinically for the treatment of spider bites (see Table 1 and Table 2, respectively). All of these antivenoms are of equine origin, and although they are effective in neutralizing scorpion and spider venoms, such animal-derived antisera suffer from significant drawbacks due to the heterologous nature of the proteins present in the antisera, which may elicit both early and late adverse reactions in human recipients [8,9]. Additionally, only a subset of the antibodies or antibody fragments present in these antivenoms have a therapeutic value since the presence of non-toxic immunogens in the venoms used for immunization may elicit therapeutically irrelevant antibodies in the immunized animal. This was demonstrated by Pucca et al., who showed that only 1%–2.5% of antibodies in equine scorpion antivenoms were able to neutralize important venom toxins [10]. Since scorpion and spider antivenoms are derived from animal serum, individual differences in the immune responses of the production animals may give rise to batch-to-batch variation [11]. Finally, due to the very minute amounts of venom that can be extracted from scorpions and spiders, production of antisera against scorpion stings and spider bites is dependent on a highly laborious venom collection process, where large numbers of spiders and scorpions need to be milked (under microscope for spiders) in order to procure enough venom for immunization [12]. These challenges warrant technological innovation, not only to obtain safer and more effective antivenoms, but also to establish more sustainable productions processes that are independent of both venoms and animals [9].

This review presents the different biotechnological trends in the development of next generation scorpion and spider antivenoms. Initially, focus will be directed towards the growing body of data on spider and scorpion toxins and proteomes, which may be harnessed for developing either recombinant or synthetic antivenoms. A comprehensive overview of the use of immunization strategies is beyond the scope of the paper, and can be found elsewhere [2]. However, the use of recombinant and synthetic toxins obtained through the use of biotechnological approaches is discussed, as well as the potential for including small molecules in future spider and scorpion antivenoms.

## 2. Current Knowledge of Toxins and Venom Proteomes

Both scorpion and spider venoms contain a range of different non-enzymatic and enzymatic toxins [39,40]. The main toxic effects of scorpion venoms are in general derived from the inhibitory actions of neurotoxins, whereas the effects of most described spider venoms originate from both enzymatic toxins and neurotoxins. The most prominent components in spider venoms are enzymatic sphingomyelinases, hyaluronidases, phospholipases, metalloproteases, serine proteases and neurotoxins [39]. Scorpion venoms on the other hand mainly consist of neurotoxins (of which particularly sodium and potassium channel targeting neurotoxins are prevalent) as well as enzymatic proteins, such as hyaluronidases and phospholipases [3], protease inhibitors, and small molecules such as histamine and serotonin [3].

Due to the very large number of spider and scorpion species, the toxin arsenal from these venomous creatures is enormous and, to a large extent, unexplored. Based on the number of species it is estimated that about 10 million spider toxins and about 100,000 scorpion toxins may exist [40]. These high theoretical numbers will naturally comprise orthologs with greater or lesser degree of sequence identity. The scorpion *Mesobuthus martensii* (Manchurian scorpion), whose genome has been sequenced, may express products from 116 neurotoxin genes, which mainly contain toxins from four groups [41]. Based on a hierarchical clustering analysis, 17 clusters with very similar toxins (closely resembling sub-family classification of the proteins) were identified within the 116 genes [41]. Taking these findings and the fact that different species can possess very similar toxins, an adjusted rough estimate of the number of distinct scorpion toxin sub-families is in the order of 100–1000. Given the much higher diversity within venomous spider families, the corresponding number of distinct spider toxin sub-families is likely to be one or more orders of magnitude higher.

As of 13 June 2016, 1483 spider toxins and 949 scorpion toxins are annotated in the UniProtKB database [42]. The vast majority of these toxins are derived from medically relevant species, possibly leaving an enormous arsenal of bioactive venom components unexplored. Although much of the exploration of spider and scorpion toxins has been triggered by an interest in using toxins for drug discovery [43] and exploring crystal structures of receptors (see e.g., [44]), this pool of structural and bioinformatics data serves as an excellent foundation for developing modern antivenoms, as several important families of toxins are represented with a significant number of entries.

**Figure 1:**
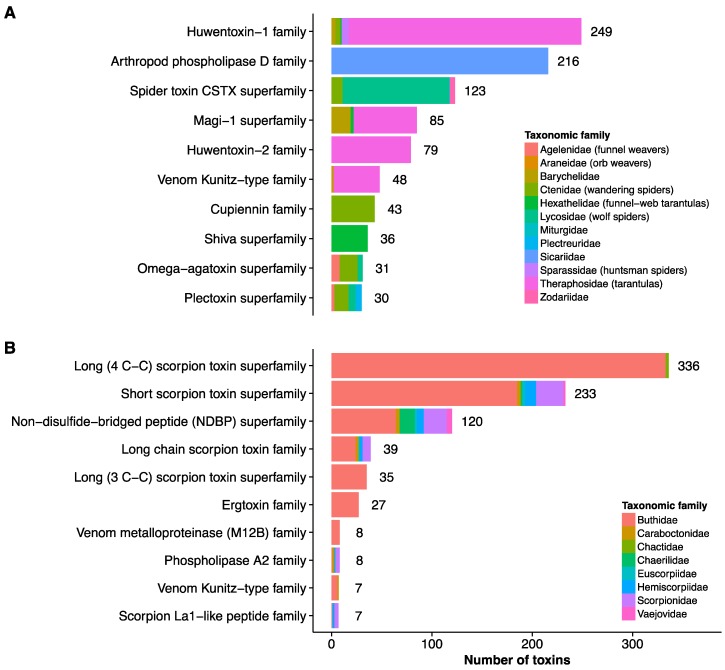
The ten protein families with the highest number of entries among the annotated (**A**) spider and (**B**) scorpion toxins in the UniProtKB database [42]. For spiders 543 toxins do not belong to any of the top ten protein families, while this number is 129 for scorpions. The bars are colored according to the taxonomic family affiliation of each toxin entry.

Our investigation shows that the majority (774 toxins) of annotated scorpion toxins (949 toxins) can be found primarily within a single scorpion family (Buthidae) and belongs to a very limited number of protein families (Figure 1B). The annotated toxins from spider venoms are more diverse both in terms of protein family and taxonomic relationship (see Figure 1A). Four spider families, namely Theraphosidae, Lycosidae, Sicariidae, and Ctenidae, are responsible for 1157 of the 1483 annotated spider toxins. The high density of annotated scorpion toxins deriving from venoms of the Buthidae family correlates with the fact that these scorpions are considered to be the most dangerous to human health among all scorpion families [2]. In contrast, the multitude of families from which annotated spider toxins originate may indicate that most studies on spider venoms have focused on finding novel toxins that may be used for drug discovery, and not on critical toxins in an envenoming case.

Deeper investigation of the UniProtKB annotated toxin dataset reveals that only 100 species of spiders and 77 scorpions contribute to the UniProtKB annotated toxin dataset. The majority of these species contribute with five or fewer toxins, indicating that only the most abundant toxins in these venoms have been characterized. The spider species with most annotated toxins are *Haplopelma hainanum* (Chinese bird spider) with 292 toxin entries, *Lycosa singoriensis* (Wolf spider) with 222 toxin entries, and *Chilobrachys guangxiensis* (Chinese earth tiger tarantula) with 104 toxin entries. The corresponding top 3 contributors for scorpions are *Mesobuthus martensii* (Manchurian scorpion) with 106 toxin entries (the only scorpion for which the genome is also available) [41], *Lychas mucronatus* (Chinese swimming scorpion) with 89 toxin entries, and *Leiurus quinquestriatus* (Egyptian scorpion) with 45 toxin entries. These numbers are likely to reflect the venom complexity of the class of creatures in general. However, the presence of a large number of entries does not necessarily indicate that the venom from a given species or the individual toxins are important from a medical point of view.

The alternative and more holistic venomics approach, aiming at constructing overviews of venom compositions on the protein family level, complements the studies of isolated toxins and may help elucidate the medical importance of different venom components. Venomics studies help generate knowledge of the abundance, toxicity, and sequence data for key toxin families, which may be harnessed for development of new cross-reacting antitoxins targeting shared features and functionalities of the toxins [45,46]. As a result of the scarcity of venoms as well as previous limitations on resolution and resolving power of separation and detection techniques, only a limited number of venomics studies determining toxin identity and abundance have been carried out [40]. A number of studies involving proteomics and/or transcriptomics have been performed for both spiders and scorpions, respectively (Table 3 and Table 4). As can be deduced from the tables, this number has been growing with increasing speed in recent years, indicating a rising interest in toxin bioactivity and venom compositions. For spiders, the main contributing species to these studies are found in the genus *Phoneutria* (wandering spiders, Ctenidae family) with ten proteomics and transcriptomics studies (see Table 3). For scorpions, the number of proteomics and transcriptomics studies (18) performed on members of the genus *Tityus* from the Buthidae family dwarfs the number of studies performed on any other genus, signifying scientific interest and indicating the importance of this genus to human health [47] (see Table 4).

Verano-Braga and coworkers provide an excellent example of a venomics study performed on the venom of *Tityus serrulatus*, in which they were able to detect the presence of 147 different proteins [111]. In terms of abundance, neurotoxins targeting sodium and potassium channels comprised 23% of the venom, while enzymatic toxins were responsible for 32% of the protein content of the venom [111]. In another study, performed by Ma et al. on the venom of *Heterometrus petersii*, 22 protein families were detected by a combined transcriptomics and proteomics approach [122], indicating high diversity in scorpion venoms. In comparison, the venom of the African *Citharischius crawshayi* (King baboon spider) was also analyzed by a venomics approach. Here, the venom was determined to be dominated by peptides in the 3–6 kDa (58%) range, followed by lower molecular weight components in the 1–3 kDa (29%) range [71], suggesting that the effects of this venom are possibly derived from smaller inhibitory toxins. A final example of a tour-de-force in proteomics is provided by Palagi and coworkers, who investigated 18 venom samples from Australian funnel-web spiders (Hexathelidae family). They detected an average of about 800 peptidic compounds in female spider venoms and approximately 400 peptidic compounds in male spider venoms with significant inter- and intra-species variation in venom compositions [40], indicating that the high toxin diversity makes venoms very complex drug targets.

**Figure 2:**
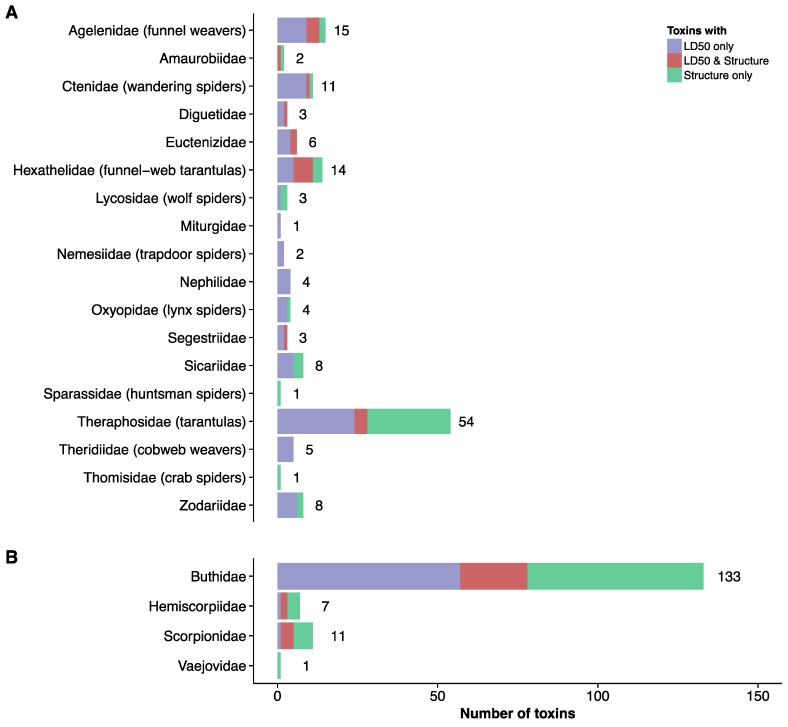
Number of toxins in each taxonomic family for which LD_50_s and/or three three-dimensional structures have been reported in the UniProtKB database [42]. (**A**) Spider toxins; (**B**) Scorpion toxins.

**Figure 3:**
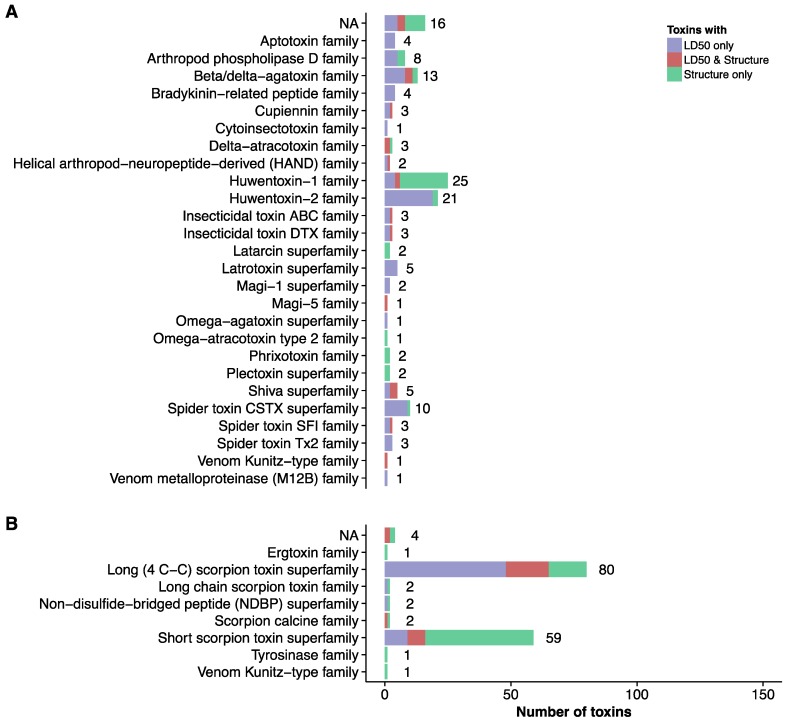
Number of toxins in each protein family for which LD_50_s and/or three three-dimensional structures have been reported in the UniProtKB database [42]. (**A**) Spider toxins; (**B**) Scorpion toxins.

In order to engage in specific toxin-directed antivenom development, a full overview of the venom is warranted. However, in addition to estimations of quantitative venom proteomes, it is highly beneficial to have information on toxin structures and estimates of toxin toxicities (e.g., LD_50_s), in order to have a full view on which toxins should be neutralized by novel antivenoms [128]. In 2016, Rodríguez-Rodríguez and coworkers employed approaches based on the Toxicity Score [46] to determine which toxins are the medically most relevant in the venoms of selected *Centruroides* scorpions [45]. This approach differentiated between medically relevant and medically irrelevant toxins, and enabled the researchers to select key toxins for antitoxin development. Furthermore, Rodríguez-Rodríguez et al. successfully employed structural analysis of complexes between toxins and antitoxins to optimize binding affinity for their inhibitors [45]. Within the annotated toxin dataset previously discussed, we find that 3D structures have been solved for a total of 63 spider toxins and 93 scorpion toxins. Moreover, LD_50_s are known for 102 spider toxins and 86 scorpion toxins. For 20 spider toxins and 27 scorpion toxins, both a 3D structure and an LD_50_ exist. The distribution of these well characterized toxin entries between the taxonomic families are visualized in Figure 2 and Figure 3.

The estimated >10 million scorpion toxins and spider toxins existing in nature [40] dwarf the number of currently annotated toxins (2432) from these creatures. Hence, a more in depth characterization would be beneficial in order to provide a better foundation for development of novel antivenoms. Furthermore, the untapped pool of highly bioactive compounds constitutes a treasure chest for toxin-derived drug discovery. Nevertheless, toxins from many of the medically important species of both spiders and scorpions have already undergone thorough investigation. The accumulated data on the structures, toxicities and abundances of these toxins represent a good foundation for developing next generation antivenoms based on biotechnological approaches and modern drug discovery techniques.

## 3. Toxin Inhibition by Small Molecules

To the best of our knowledge, no small organic molecule has ever been tested for spider or scorpion toxin neutralization in a clinical setting. Compared to snake venoms, limited research efforts have focused on the discovery of small-molecule drugs targeting specific toxins in scorpion and spider venoms. Only a handful of compounds has been reported to have neutralizing effects on scorpion or spider venom components (Table 5).

The first reported small molecule that was investigated in relation to inhibition of scorpion toxins was heparin. Using turbidometric methods, heparin has been shown to inhibit hyaluronidases isolated from various scorpion species, such as the Chinese red scorpion (*Buthus martensi*) [129] and the Indian black scorpion (*Palamneus gravimanus*) [130]. In addition, heparin has been shown to inhibit the hyaluronidase activity of venom from *Heterometrus fulvipes*, a species of the giant forest scorpions [140]. However, most studies on the effect of heparin on spider venoms demonstrate no inhibitory effect on venom-induced necrosis [141]. In contrast, surgical excision or administration of effective antivenom has been shown to be more efficacious [142]. However, it has been suggested that administration of heparin alongside other treatments, such as steroids, hyperbaric oxygen treatments, experimental antivenom, and/or surgical excision, may be beneficial in the treatment of bites from Loxosceles spiders [143]. 

Neutralization studies on venom from the Brazilian yellow scorpion (*Tityus serrulatus)* with Aristolochic acid demonstrate that this polyphenolic compound has the ability to decrease lethality in mice [132]. Although focus in this study was on hyaluronidase enzymes, direct inhibition of hyaluronidases was not tested. Other studies have shown, however, that Aristolochic acid has an inhibitory effect on phospholipases A_2_ (PLA_2_s), which are found in scorpion venoms [120,132]. To date, no inhibition studies with Aristolochic acid on PLA_2_s from spider venom have been reported, despite the presence of PLA_2_s in arachnid venoms.

**Figure 4:**
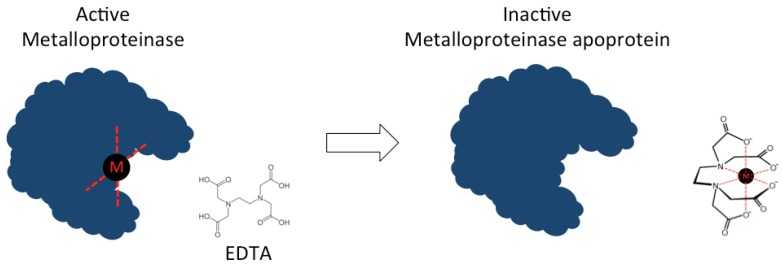
Mechanism of inactivation of metalloproteases. The chelation agent EDTA chelates metal ions and scavenges these from active metalloproteases leaving behind the inactive metalloprotease apoprotein.

The bivalent metal ion chelators, EDTA and 1,10-phenanthroline, both function as metalloprotease inhibitors (see Figure 4), and several studies indicate that both compounds are capable of neutralizing spider and scorpion venoms by inhibiting various venom components [133,134]. EDTA has been shown to inhibit PLA_2_ isolated from the scorpion *H. fulvipes* [140], as well as a range of different proteinases isolated from the venom of the scorpion *Isonietrus vittatus* from Pakistan [135]. Moreover, both EDTA and 1,10-phenatroline inhibit heminecrolysin, which is the enzyme responsible for the main pathological effects in envenomation by the scorpion *Hemiscorpius lepturus* [133]. Heminecrolysin is completely inhibited in vitro by EDTA at a concentration of 5 mM, and 50% inhibited by 1,10-phenantroline at a concentration of 2.5 mM [133].

Apart from the inhibition of individual venom components, EDTA has also been shown to inhibit the proteolytic activity of the venom from *T. serrulatus* on fibrinogen [136,139]. A similar inhibition of proteolytic activity on fibrinogen has also been observed in vitro for EDTA (concentration of 2 mM) and 1,10-phenanthroline (concentration of 3 mM) on *Loxosceles intermedia* (Brown spider) venom [134]. Finally, both EDTA and 1,10-phenanthroline, at a concentration of 5 mM each, have been shown to specifically inhibit metalloproteases isolated from *Hippasa partita* venoms [138], whilst EDTA (1 mM) alone has proven to block metalloproteases from *Parawixia bistriata* (Araneidae genus) [137].

In addition to the examples discussed above, a number of other molecules have shown neutralizing effects on toxins similar to those found in spider and scorpion venoms. As an example, a recent study by El-kik et al. (2013) has indicated the ability of the poly-anionic compound suramin to antagonize the cytotoxic and enzymatic effects of crude venom from the bee, *Apis mellifera* [144]. Interestingly, suramin has previously been indicated to inhibit the myotoxic actions of PLA_2_s in snake venoms [128]. Therefore, it would not be surprising if suramin showed the same inhibitory effects on scorpion and spider venoms containing these enzymes in abundance [39,145]. In a similar way, a large number of other compounds that have shown neutralizing effect against snake venom toxins could potentially also inhibit toxins from spiders and scorpions (particularly PLA_2_s) [128]. Nevertheless, further experimental investigation is needed to determine such potential effects.

In conclusion, no small molecules targeting toxins from scorpion or spider venoms are in use in the clinic. Although only few small molecules have been investigated, a range of compounds acting on similar toxins from snakes has been discovered [128]. The potential of using such molecules in future spider bite or scorpion envenoming therapy could be pursued with the aim of developing compounds with improved stability and a large volume of distribution. Such properties may be of particular benefit for targeting locally acting toxins in distal tissue, such as muscle tissue in arms and legs, which are notoriously difficult to neutralize with traditional antivenoms. However, further research is needed to explore the feasibility of this approach.

## 4. Research Efforts within Antibodies and Antibody Fragments

Modern biotechnological techniques for the generation, isolation, and production of monoclonal antibodies (mAbs) or antibody fragments have recently been applied in the experimental development of next generation antivenoms against scorpion stings and spider bites (see Table 6, Table 7, and Table 8) [9]. The first reported neutralizing mAb in this field (mAb 4C1) was directed against the venom of the *Androctonus australis hector* (Aah) scorpion [146], and it was generated using hybridoma technology [147]. The toxicity of *A. australis hector* venom is derived from three small neurotoxins belonging to two distinct structural and immunological groups (Group 1: Aah I and Aah III, and Group 2: Aah II), and mAb 4C1 showed affinity for Aah II with a K_d_ of 0.8 nM [146]. Another antibody, mAb 9C2, was generated against Aah I with a K_d_ of 0.15 nM, and it showed to be cross-reactive with other toxins of the same group, having K_d_s of 1.5 nM for Aah III and 24 nM for Aah IV [148]. In mice, 1 mg of mAb 4C1 neutralizes 30,000 LD_50_s of Aah II, and 1 mg of mAb 9C2 neutralizes 1500 LD_50_s of Aah I. When tested against whole venom, both mAbs were capable of neutralizing 40 LD_50_s [146,148]. Moreover, 1 mg of the mixture of 9C2 and 4C1 was able to neutralize 71 LD_50_ of the venom [148], providing an excellent example of how selected mixtures of antibodies can be used to neutralize selected medically relevant toxins, thereby abrogating the toxicity of whole venom [46].

Similarly, the neutralizing mAb BCF2 was generated against toxins from scorpions of the genus *Centruroides* [152]. The venoms of the three main species of *Centruroides* scorpions contain toxins of high similarity (Css2 from *C. sufusus suffusus*, Cn2 from *C. noxius*, and CII1 and CII2 from *C. limpidus limpidus*), and all bind to Na^+^ ion channels in excitable cells, modifying ion channel activation and/or inactivation. BCF2 was directed against Cn2 from *C. noxius*, and 1 mg of this mAb was capable of neutralizing 32 LD_50_s of the purified toxin and 28 LD_50_s of whole venom in mice [153]. Moreover, the antibody was tested for cross-reactivity against the venoms of *C. limpidus limpidus*, *C. limpidus tecomanus*, *C. limpidus acatlanesis*, *C. suffusus suffusus*, *C. infamatus infamatus*, *C. elegans* and *T. serrulatus* in ELISA plates. Despite a high degree of similarity between toxins from related scorpion families, a clear strong signal was only detected against venom components of *C. limpidus limpidus*, *C. limpidus tecomanus* and *C. limpidus acatlanesis*. Unfortunately, the neutralization capacity of BCF2 against venoms from these species was not tested in these studies.

The first reported mAb against a spider toxin was Li mAb(7), also obtained from a hybridoma cell line [149]. Li mAb(7) was directed against venoms of spiders from the *Loxosceles* genus, commonly known as recluse or brown spiders. The toxic effects of brown spiders are elicited by proteins of the sphingomyelinase D (SMase D) family, which cause local effects at the bite site, such as edema, local hemorrhage, and necrotic lesions, and systemic effects, such as hemolysis, thrombocytopenia, and renal failure in some occasions. Li mAb(7) is capable of preventing dermonecrotic activity in mice by targeting SMase D of *L. intermedia*. Nevertheless, the antibody did not show cross-reactivity with venoms from other *Loxosceles* species [149]. Recently, a new murine mAb (LiD1mAb16) able to recognize a highly conserved epitope in the SMases D of the three major medically important species of *Loxosceles* spiders was generated by the same procedure. However, in vivo assays are only reported for this mAb against the recombinant toxin and not whole venom [151].

**Figure 5:**
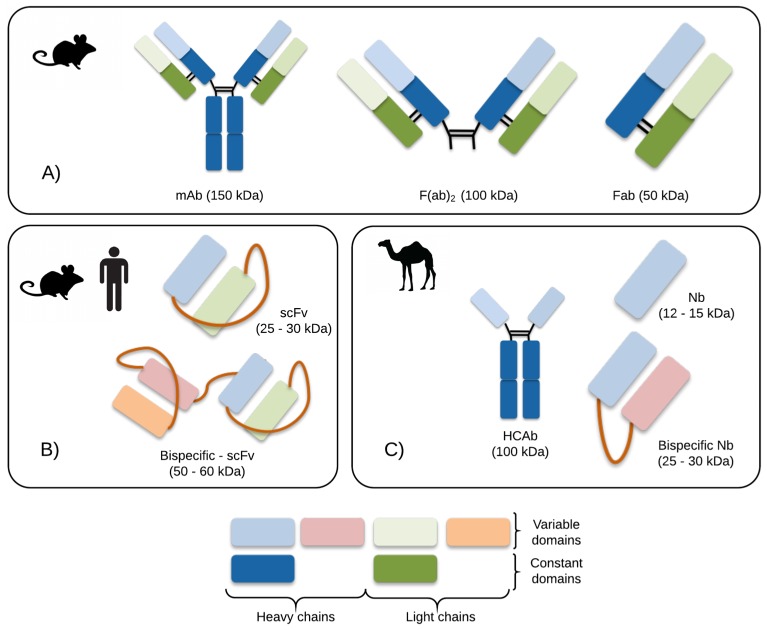
Schematic representation of the antibody formats mentioned in the text. (**A**) Whole mAb and mAb fragments obtained after enzymatic cleavage, usually derived from hybridoma cell lines; (**B**) Recombinant antibody molecules of human or murine origin, usually selected from synthetic libraries by phage display selection; (**C**) Camelid heavy chain antibody (HCAb) and derived formats*.*

The ability to clone and synthetically produce antibodies opened the possibility for replacing hybridoma cell lines for heterologous expression systems and also exploring other antibody formats with potentially optimized pharmacokinetic properties and enhanced shelf lives (Figure 5). As an example, single-chain variable fragments (scFvs) based on the mAbs 9C2 and 4C1 were developed and expressed in *E. coli*, and the resulting scFvs have K_d_s in the same nanomolar range as the intact immunoglobulins [154,156]. In vivo, scFv4C1 displayed a protective capacity of 4000 LD_50_s of Aah II per mg of scFv [154], and scFv 9C2 showed a protective capacity of 6600 LD_50_s of Aah I per mg of the antibody fragment [156]. Due to their reduced size (Mw around 30 kDa), scFvs have been argued to have several potential advantages, such as increased diffusion rates, increased tissue penetrability, increased biodistribution, and decreased immunogenicity. Since toxins are rapidly absorbed and distributed, scFvs are potentially optimal molecules for neutralizing the effects of animal toxins [175]. In order to increase efficacy by exploiting avidity, scFvs can be joined together by a short linker, creating bivalent molecules able to bind two different antigens simultaneously. The scFvs 4C1 and 9C2 were used as building blocks to design a tandem-scFv comprising the two scFvs fused together at the genetic level (T94H6). This tandem-scFv showed K_d_s of 0.1 nM against Aah I and 1 nM against Aah II. When tested in vivo, all mice co-injected with T94H6 and 3 LD_50_s of Aah I survived, whereas the tandem-scFv only protected 50% of the mice challenged with 3 LD_50_s of Aah II [161].

**Figure 6:**
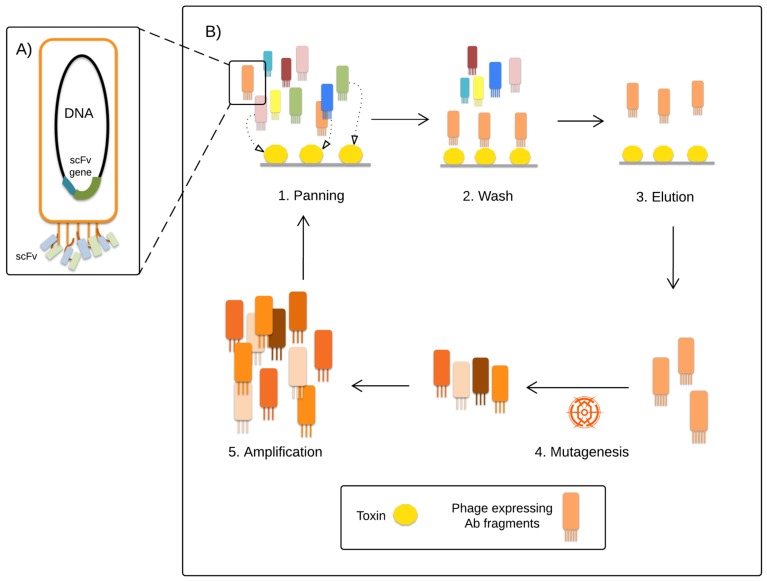
Schematic representation of a directed evolution approach by phage display selection coupled to mutagenesis for discovery of high affinity antibody variants. (**A**) Representation of a phage particle encoding and displaying scFv molecules on its surface. (**B**) Phage particles displaying a library of antibody fragments are panned against the target toxin (1). Strongly binding phages remain bound to the target, while non-binding phages are washed away (2). Binding phages are eluted and (3) submitted to mutagenesis, usually by error prone PCR or chain shuffling, with the intention of obtaining phage particles with enhanced affinities towards the target (4). The obtained mutants are amplified in *E. coli* and submitted to new panning rounds (5). After a few iterative cycles, the most strongly binding phages are eluted, and their DNA is sequenced to reveal which antibody fragments bound most strongly to the target.

A major breakthrough in antibody technology was achieved when McCafferty et al. succeeded in expressing the antibody V domains on filamentous M13 phages, allowing for rapid screening of antibody genes via phage display selection (Figure 6) [176]. Using this technique, scFvs based on the mAb BCF2 were developed using error-prone PCR to introduce mutations in the CDR regions. Improved variants were selected by phage display, and two of these, scFv G5 and scFv B7 (containing two and one mutations, respectively), showed enhanced stability and a higher affinity for the toxin (Kds of 0.43 nM and 0.71 nM, respectively) compared to the parental mAb. However, they still failed to neutralize the toxin [159]. Subsequently, in order to overcome this weakness, the mutations contained in G5 and B7 were introduced in a scFv triple mutant, which showed a Kd of 75 pM and increased stability. In mice, 1 mg of the dimeric construct was able to neutralize 400 LD_50_s of Cn2 [159].

One of the scarce examples of antibody fragments against spider toxins is the Fragment antigen-binding (Fab) FM1, directed against α-latrotoxin from the venom of spiders of the genus *Lactrodectus*, which are commonly known as widow spiders [150]. The venom of widow spiders causes potent neurotoxic effects by destabilizing presynaptic membranes of nerve terminals, which leads to Ca^2+^ influx and massive release of neurotransmitters. FM1 was isolated from a phage display library constructed with the antibody repertoire of immunized mice and showed an affinity for the toxin in the low nM range. When mice were treated with one LD_100_ of whole venom, 20 μg of purified FM1 prevented lethality in 100% of the cases [150].

Another important antibody format is the single variable domain, consisting of only the antibody heavy chain (V_H_H). These V_H_H fragments (popularly termed “nanobodies”, abbreviated “Nb”) can be isolated from the immune system of camelids and sharks [128,177]. With a molecular weight of 15 kDa, nanobodies represent the smallest, intact, natural antigen-binding fragments known, and they have the beneficial properties of good stability (ex vivo), high level of expression in microbial systems, high solubility, good specificity, and high affinity for their respective antigens [178,179]. Although nanobodies are rapidly cleared from the blood [180], their low molecular weight may be advantageous for reaching the toxins having been distributed into organs and deep tissue upon a scorpion sting or spider bite. Moreover, nanobody-toxin complexes remain below the renal cut-off (60 kDa), thus enabling their excretion by renal clearance in envenomed victims [168].

In 2009, a large V_H_H library from the lymphocytes of an immunized dromedary was generated and screened by phage display selection against Aah II [164]. One particular nanobody (NbAahII10) showed an extraordinarily high neutralizing capacity (37,000 LD_50s_ of Aah II per mg of nanobody). This represents a considerable accomplishment of paramount importance in the field of antibody fragments [164]. In 2010, the same group selected the nanobody NbAah’F12 against Aah I by a similar procedure, and this nanobody provided full protection in mice challenged with 100 LD_50_s of the toxin [165]. In the same study, a bispecific nanobody of 29 kDa directed against Aah I/Aah II, NbF12-10, was reported to neutralize 5 LD_50_s of whole venom and restoring the heart rate dysfunction induced by Aah envenoming in mice [165].

Thus far, none of the aforementioned mAbs or antibody fragments have been of human origin. Therefore, they come with a risk of eliciting undesired adverse reactions in human recipients. An attempt to overcome this issue is exemplified by the development of chimeric human antibody constructs, which are obtained by joining the variable region genes of an antibody molecule produced from a myeloma cell line with human immunoglobulin constant region genes [181]. The non-human regions of the resulting mAb are reduced while the pharmacokinetic properties remain unaffected. The chimeric Fab, chFab-BCF2, comprises the variable regions of the murine mAb BCF2 and human constant regions. In vitro, chFab-BCF2 showed an IC_50_ of 3 nM, which is in the same range as the IC_50_ of the whole mAb. When tested in mice, 3.75 μg of the chimeric construct neutralized one LD_50_ of the toxin, and 6.25 μg neutralized one LD_50_ of whole venom [157].

Humanization procedures have been developed for murine mAbs and its diverse formats [182,183], but humanization is a complex procedure, often accompanied by a decrease in affinity and neutralizing capacity of the molecules [182]. Therefore, mAbs should ideally be of human origin to be included in antivenoms. In the last decade, a few mAbs directed against scorpion toxins have been isolated from human phage display libraries (see Table 8). However, no mAbs of human origin against spider toxins have yet been reported.

In 2005, Riaño-Umbarila et al. isolated scFvs 3F and C1, two recombinant human antibody fragments capable of neutralizing the toxic effects of the toxin Cn2 and whole venom of *C. noxius* [162]. In the same study, scFv 3F was matured by three cycles of directed evolution to obtain scFv 6009F, with a K_d_ of 0.41 nM for Cn2. In vivo, scFv 6009F neutralized 2 LD_50_s of the Cn2 toxin when molar ratios of 1:10 and 1:2 toxin-to-antibody fragment were injected [162]. Some years later, the same group showed that scFv 6009F was cross-reactive against toxins Css2, Cn3, and Css4, suggesting that the fragment was able to recognize a conserved epitope important for neutralizing the toxin [166]. Subsequently, additional maturation cycles were performed with the parental scFv against the toxin Css2 from *C. suffusus*, leading to the isolation of scFv 9004G. scFv 9004G showed a K_d_ of 0.81 nM for Css2, and was able to provide protection in mice challenged with 1:10 molar ratio of toxin-to-antibody fragment [166]. With the aim of obtaining a molecule capable of simultaneously neutralizing Cn2 and Css2, the important mutations for toxin recognition were identified in both scFv 6009F and scFv 9004G, and the best properties were merged to generate a third scFv, named LR. scFv LR had a K_d_ value of 0.02 nM for Cn2 and 0.09 nM for Css2, and showed excellent stability in vitro when submitted to strong denaturing conditions. In vivo, all mice survived when treated with 1:10 molar ratios of toxin-to-antibody fragment for Cn2 or Css2, and the antibody fragment was able to provide full protection when 3 LD_50_s of whole venom of *C. suffusus* or *C. noxius* were administered at a molar ratio of 1:3 [166]. scFv LR and scFv 6009F were both further optimized in subsequent studies through directed evolution and further biopanning of Diabody 6009F (a dimerized version of scFv 6009F). This led to the identification of a new mutation (E43G), which provided increased functional stability and neutralization capacity for the optimized versions, Diabody D4 and scFv LER, compared to Diabody 6009F and scFv LR [170]. An additional scFv, named 202F, was derived from scFv C1 after 3 cycles of directed evolution against toxin CII1 from *C. limpidus*, and it neutralized one LD_50_ of both Cn2 and CII1 toxins [173]. With the aim of improving the neutralization capacity of scFv 202F, the same group generated a new antibody fragment, named scFv RU1, through incorporation of two different mutations. The scFv neutralized one LD_50_ of CII1 toxin, two LD_50_s of Cn2 toxin, and 3 LD_50_s of fresh whole *C. noxius* venom [174]. To expand the neutralization capacity toward additional scorpion toxins, scFv RU1 was subjected to in vitro maturation followed by phage display selection in order to identify mutations yielding cross-reactive scFvs with uncompromised affinity towards the toxins. By employing this approach and combining different identified mutations, the scFv ER-5 was obtained. scFv ER-5 recognized toxins Css2, Css4, Cn2, and CII1, and neutralized one LD_50_ of both Cn2 and CII1 toxins, and two LD_50_s of whole *C. suffusus* venom [45], which demonstrates the power of phage display technology coupled to semi-rational approaches for affinity optimization.

In the case of *T. serrulatus*, a scorpion responsible for most of the severe cases of scorpion envenomation in Brazil, a neutralizing scFv (scFv 15e) was isolated by phage display selection from a human library. The most toxic components of the venom of *T. serrulatus* are neurotoxins that exert their effect by binding to voltage-gated Na^+^ ion channels. Neurotoxins are classified into α-toxins (Ts3 and Ts5) and β-toxins (Ts1 and Ts2). scFv 15e is directed against Ts1, the main toxic component of *T. serrulatus* venom, and also recognizes toxins from *T. cambridgei* and *T. packyurus*, two related scorpions from the same genus [167]. The neutralization capacity of scFv 15e against Ts1 was very limited, as 12 out of 16 mice challenged with one LD_50_ of the toxin survived [167]. Currently, optimized variants of scFv 15e are being obtained using a directed evolution approach coupled to phage display with the aim of enhancing its neutralization capacity, but successful results have yet to be reported [167]. 

Another relevant example of a human scFv is Serrumab, which is directed against toxins in the venom of *T. serrulatus.* Serrumab was isolated after four rounds of phage display selection from a synthetic human library against whole venom of the scorpion. In vitro, Serrumab neutralized the toxins Ts1 and Ts2 (β-toxins), but undetectable or low reactivity was reported against Ts3 and Ts5 (α-toxins). In vivo, the increase in the plasma levels of urea, creatinine, and neutrophil recruitment associated to envenomation was not observed in mice treated with Serrumab, in contrast to animals that received venom alone [171]. Furthermore, Serrumab showed cross-reactivity against Css II from *C. suffusus suffusus* and Lqh III from *L. quinquestriatus hebraeus*. Nevertheless, in vivo assays using isolated toxins have not yet been performed due to the difficulty of obtaining sufficient quantities of toxins [172]*.*

The highlighted examples above display some trends in the development effort within spider and scorpion envenomation therapies. Increasing interest and efforts exist within the generation of recombinant, human antibody fragments using phage display technology [9]. The benefits of using phage display technology include the possibility of modifying the antibodies or fragments by genetic engineering techniques or affinity maturation by directed evolution. In addition, manufacturing processes for antibodies and antibody fragments have undergone a major development in the past 30 years with production cost of antibodies having decreased significantly [184,185]. Developments in both antibody technologies and manufacturing technology may indeed be the key to enable production and distribution of antibody-based envenomation therapies to poor, rural parts of the tropical world, where the cost of medicine may be an inhibiting factor for treatment. In comparison to scorpions, the field of antibody-based antitoxins against spider toxins is less developed. Reasons for this may include the minor severity or lower prevalence of spider bites in most of the world, and the difficulty of obtaining sufficient quantities of medically important spider venoms.

## 5. Next Generation Immunization Strategies

**Figure 7:**
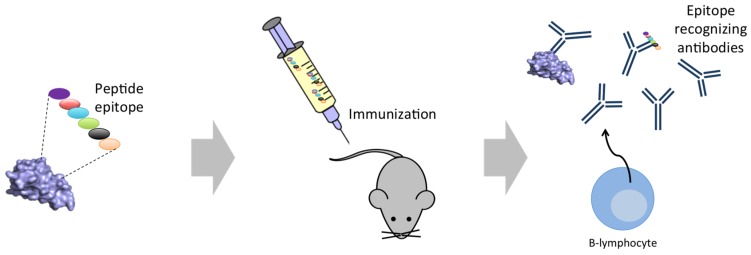
Schematic representation of the use of peptidic epitopes for immunization. A peptide containing the most reactive epitope sequence(s) from selected toxin(s) is constructed and used for immunization of mice. In a successful immunization, the antibodies raised will not only target the peptide, but also the parent toxin(s).

A complementary approach to using recombinant antibodies or antibody fragments is the use of recombinant toxins or synthetic peptides for animal immunization procedures (Figure 7). Such approaches may provide a path to obtaining antivenoms with higher titers of antibodies against the relevant toxins and low titers against medically unimportant venom components. Within spider and scorpion antivenoms, efforts have been made with both strategies (Table 9 and Table 10). However, in contrast to the field of snakebite antivenoms, the use of DNA immunization techniques has not been explored [128].

Within scorpion antisera, the first use of a synthetic peptide for immunization was reported in 1986, where Bahraoui et al. conjugated a nine amino acid long epitope from the AaH2 toxin from *A. australis* to albumin. The anti-peptide serum raised in rabbits against the albumin-AaH2 epitope was able to neutralize the natural toxin, but no results against the crude venom were reported [190]. In 1999, Calderon-Aranda et al. engineered a peptide from the *Centruroides noxius* Cn2 toxin, containing continuous and discontinuous epitopes, by linking the N- and C-terminus of the resulting peptide by a disulfide bridge. The anti-peptide antiserum produced in rabbits could neutralize 39.5 LD_50_ of Cn2 per mL of serum [194]. 

In 2000, Guatimosim et al. reported the use of a non-toxic, recombinantly expressed version of the immunogenic protein (TsNTxP) from *T. serrulatus* venom for immunization of rabbits [195]. 1 mL of the raised antiserum was able to protect rabbits injected with 20 LD_50_ of the whole venom. The TsNTxP-derived protein was produced as a fusion protein with a maltose-binding protein, in order to avoid degradation by the molecular machinery of *E. coli* cells [195]. 

In 2004, Machado de Ávila et al. prepared an immunogen consisting of the most toxic fraction (TstFG50) of the *T. serrulatus* venom conjugated to bovine serum albumin (BSA). Mice immunized with TstFG50-BSA showed full protection when challenged with 2 LD_50_s of TstFG50 [205]. In a more recent study, a SPOT mapping method was used to identify discontinuous epitopes of TsNTxP, and the most reactive peptide sequences were combined on a linear strand (GREGYPADGGGLPDSVKI). This linear strand was used for immunization of mice, which were fully protected when challenged with 1.75 LD_100_s of *T. serrulatus* whole venom [204].

Also using the SPOT method, Alvarenga et al. mapped three antigenic regions on TsNTxP and one epitope on TsIV, the latter of which is the most lethal venom component of *T. serrulatus* belonging to the alpha-type toxin family. Antibodies from antiserum raised against these four peptides were conjugated to keyhole limpet hemocyanin (KLH), and 1 mL of the anti-peptide serum was able to neutralize 13.5 LD_50_s of whole venom from *T. serrulatus* in vitro. Apart from showing high reactivity against the crude venom of *T. serrulatus*, the anti-peptide antibodies in the serum bound moderately to the venoms of *T. bahiensis*, *T. cambridgei*, *T. stigmurus*, and venoms from the *Centruroides* genus. Therefore, this study showed that antibodies generated against peptides derived from the toxins of one scorpion can cross-react and neutralize venoms from different scorpion species, providing an alternative strategy for the preparation of polyspecific scorpion antivenoms [198].

Within spider antivenoms, the use of both recombinant and synthetic toxins for immunization has been explored. In 2003, Araujo et al. constructed a recombinant fusion protein between a protein homologous to a dermonecrotic toxin from *L. intermedia* and beta-galactosidase. The protein was expressed in *E. coli*, and subsequently used as antigen for the immunization of rabbits and mice. The immunized rabbits showed prolonged in vivo protection against the whole venom, and the mice achieved full protection against 2.5 LD_50_s of whole venom [186]. In 2013, Mendes et al. constructed a chimeric protein containing three epitopes of LiD1, a dermonecrotic toxin present in the venom of *L. intermedia*. When rabbits were immunized with the recombinant chimeric protein (rCpLi), they were able to elicit a protective immune response against whole venom from both *L. intermedia* and *L. gaucho*, comparable to rabbits immunized with the natural toxin [189]. 

In 2009, Comis et al. chemically synthesized the 42 amino acid long robustoxin from the venom of *Atrax robustus*, and used this toxin for immunization of *Macaca fascicularis* monkeys. The toxin was prepared without establishing the disulfide bridges that are essential for toxicity, and co-polymerizing the toxin with keyhole limpet haemocyanin. Immunized monkeys survived when challenged with a lethal dose of the crude venom, having a full recovery with only minor symptoms of envenoming [188].

Although the use of recombinant toxins or synthetic peptides for immunization of animals does not radically change the nature of antivenoms, several potential benefits may be reaped. First of all, it excludes the need for laborious venom extraction, where either large numbers of spiders need to be farmed or caught and milked under a microscope, or where electrical stimulation methods are needed for collection of scorpion venoms [12]. Independence of venoms may not only help reduce cost, but it may also yield a much less hazardous production process, remove the risk and impact of potential diseases among spiders or scorpions used for venom collection, and remove the effect of individual venom variability among specimens used for venom collection. Moreover, the possibility of only using the medically relevant toxins or peptide epitopes for immunization may yield better titers of therapeutically active antibodies which, in turn, may lead to more potent antivenoms. In addition to higher efficacy, more potent antivenoms may elicit fewer side effects, since less antivenom is needed to treat the envenomed victim [128]. However, limitations to these strategies include the inability of some heterologous expression organisms to produce recombinant toxins with correct disulphide bonding and protein structure, and the fact that linear peptides may not always yield therapeutically active antibodies when used for immunization [2].

## 6. Future Perspectives

The array of analytical tools for analysis of venoms and toxins may possibly lay the foundation for introducing new innovations into the field of scorpion and spider antivenoms. Given that less medically relevant toxins in scorpion and spider venoms are essential to neutralize compared to snake venoms [9], and given the more laborious venom extraction procedures required for current scorpion and spider antivenom production, introduction of recombinant production methods for oligoclonal human antibodies or fragments thereof may indeed be a promising possibility, which is likely to be both feasible, cost-competitive [185], and which may yield more efficacious serum-free antivenoms with better safety profiles [11,128]. Coupled to powerful discovery platforms, such as phage display selection or the use of humanized animals, recombinant expression technologies could revolutionize the field of antivenoms by delivering safer, more efficacious therapies that are affordable for the healthcare systems of the developing world. However, in order for such a technological breakthrough to happen, more research efforts need to be directed towards scorpion and spider venomics, antitoxin discovery, and novel expression technologies.

Hopefully, the future may generate novel therapies that can help combat the neglected area of scorpion and spider envenomings, causing much mortality and morbidity among people living the regions affected by this burden.

## Figures and Tables

**Table 1:** Antivenoms on the market for treatment of spider bite envenomings.

Product Name	Producer	Country	Type	Spiders	Link Reference
Funnel web spider antivenom	CSL Ltd.	Australia	Equine F(ab’)_2_	Hexathelidae family (funnel-web spiders)	[13]
Red Back Spider antivenom	CSL Ltd.	Australia	Equine F(ab’)_2_	*Latrodectus hasselti* (redback spider)	[14]
Aracmyn	Instituto Bioclon	Mexico	Equine F(ab’)_2_	*Latrodectus mactans* (black widow spider), *Loxosceles* spp. (recluse spiders)	[15]
Reclusmyn	Instituto Bioclon	Mexico	Equine F(ab’)_2_	*Loxosceles* spp. (recluse spiders)	[16]
Soro antiarachnidico	Instituto Butantan	Brazil	Equine F(ab’)_2_	*Loxosceles* spp. (recluse spiders), *Phoneutria* spp. (Brazilian wandering spiders)	[17]
Anti Latrodectus antivenom	Instituto Nacional de Biologics A.N.L.I.S.	Argentina	N/A	*Latrodectus mactans* (black widow spider)	[18]
Suero antiloxoscélico monovalente	Instituto Nacional de Salud, Perú	Perú	Equine IgG	*Loxosceles* spp. (recluse spiders)	[19]
Soro Antilatrodéctico	Instituto Vital Brazil	Brazil	Equine F(ab’)_2_	*Latrodectus mactans* (black widow spider)	[20]
Antivenin (*Latrodectus mactans*)	Merck Sharp and Dohme International	USA	Equine IgG	*Latrodectus mactans* (black widow spider)	[21]
SAIMR Spider Antivenom	South African Vaccine Producers	South Africa	N/A	*Latrodectus indistinctus* (black button spider)	[22]

**Table 2:** Antivenoms on the market for treatment of scorpion sting envenomings.

Product Name	Producer	Country	Type	Scorpions	Link Reference
Suero antialacran	BIRMEX	Mexico	N/A	*Centruroides* spp. (bark scorpions)	[23]
Suero antiescorpiónico	Centro de Biotecnologia de la Universidad central de Venezuela	Venezuela	Equine F(ab’)_2_	*Tityus* spp. (thin-tailed scorpions)	[24]
Le sérum antiscorpionique (monovalent)	Institut Pasteur d’Algerie	Algeria	N/A	*Androctonus australis* (fat-tailed scorpion), B*uthus occitanus* (common yellow scorpion), *Androctonus crasicauda* (Arabian fat-tailed scorpion)	[25]
Scorpion antivenom	Institut Pasteur du Maroc	Morocco	Equine F(ab’)_2_	*Buthus occitanus* (common yellow scorpion), *Androctonus mauritanicus* (Moroccan fat-tailed scorpion)	[26]
Le sérum antiscorpionique	Refik Saydam Hygiene Center	Turkey	Equine	*Androctonus crassicauda* (Arabian fat-tailed scorpion), *Leiurus quinquestriatus* (Israeli yellow scorpion)	[27]
Alacramyn	Instituto Bioclon	Mexico	Equine Fab	*Centruroides* spp. (bark scorpions)	[28]
Soro antiarachnidico	Instituto Butantan	Brazil	Equine F(ab’)_2_	*Tityus* spp. (thin-tailed scorpions)	[17]
Soro antiescorpionico	Instituto Butantan	Brazil	Equine F(ab’)_2_	*Tityus bahiensis* (black scorpion), *Tityus serrulatus* (Brazilian yellow scorpion)	[29]
Soro antiescorpiônico	Instituto Vital Brazil	Brazil	Equine F(ab’)_2_	*Buthus occitanus* (common yellow scorpion)	[20]
Polyvalent Scorpion Antivenom	National Antivenom and Vaccine Production Center	Saudi Arabia	Equine F(ab’)_2_	*Leiurus quinquestriatus* (Israeli yellow scorpion), *Androctonus crassicauda* (Arabian fat-tailed scorpion), *Buthus arenicola*, *Buthus mimax*, *Buthus occitanus* (common yellow scorpion), *Androctonus amoreuxi* (fat-tailed scorpion)	[30]
Le sérum antiscorpionique	Pasteur Tunis	North Africa	Equine F(ab’)_2_	*Androctonus australis* (fat-tailed scorpion), *Buthus occitanus* (common yellow scorpion)	[31]
Monovalent Scorpion Antivenom	Razi Vaccine and Serum Research Institute	Iran	Equine	N/A	[32]
Polyvalent Scorpion Antivenom	Razi Vaccine and Serum Research Institute	Iran	Equine	*Androctonus crasicauda* (Arabian fat-tailed scorpion), *Hemiscorpius lepturus*, *Hottentotta saulcyi*, *Hottentotta schach*, *Mesobuthus eupeus*, *Odontobuthus doriae*	[32]
Scorpifav	Sanofi Pasteur	North Africa and Middle East	Equine F(ab’)_2_	*Androctonus australis* (fat-tailed scorpion), *Leiurus quinquestriatus* (Israeli yellow scorpion), *Buthus occitanus* (common yellow scorpion)	[33]
SAIMR Scorpion Antivenom	South African Vaccine Producer	South Africa	Equine	*Parabuthus transvaalicus* (dark scorpion)	[22]
Scorpion antivenom Twyford	Twyford Pharmaceuticals	North Africa	N/A	*Androctonus australis* (fat-tailed scorpion), *Buthus occitanus* (common yellow scorpion), *Leiurus quinquestriatus* (Israeli yellow scorpion)	[34]
Purified Polyvalent anti-scorpion serum	VACSERA	Egypt	Equine F(ab’)_2_	*Leiurus quinquestriatus* (Israeli yellow scorpion), *Scorpio maurus* (large-clawed scorpion), *Androctonus crasicauda* (Arabian fat-tailed scorpion), *Buthus occitanus* (common yellow scorpion)	[35]
Scorpion Venom Antiserum	Vins Bioproducts Ltd.	India	Equine IgG	*Leiurus quinquestraitus* (Israeli yellow scorpion), *Androctonus amoreuxi* (fat-tailed scorpion)	[36]
Soro Antiescorpiônico (FUNED)	Fundação Ezequiel Dias	Brazil	Equine F(ab’)_2_	*Tityus serrulatus*, (Brazilian yellow scorpion)	[37]
Anti-scorpion Venom Serum	Haffkine Bio-Pharmaceutical Corporation Ltd.	India	Equine IgG	*Buthus tamulus* (red scorpion)	[38]

**Table 3:** Proteomics and transcriptomics studies performed on spider venoms.

Family	Genus	Species	Prot.	Tran.	Reference
Agelenidae	*Agelena*	*A. orientalis* (funnel weaver spider)	√	-	[48]
Araneidae	*Araneus*	*A. ventricosus* (Chinese orb-weaving spider)	√	√	[49]
Barychelidae	*Trittame*	*T. loki* (brush-foot trapdoor)	√	√	[50]
Ctenidae	*Cupiennius*	*C. salei* (tiger wandering spider)	√	-	[51]
	*Phoneutria*	*P. boliviensis* (male) (Brazilian wandering spider)	√	-	[52]
		*P. boliviensis* (female)	√	-	[52]
		*P. nigriventer* (Brazilian wandering spider)	√	√	[53,54]
		*P. nigriventer* (Minas Gerais, Brazil)	√	-	[55]
		*P. pertyi*		√	[53]
		*P. keyserlingi* (Minas Gerais, Brazil)	√	-	[55]
		*P. reidyi* (Amazonas, Brazil) (Brazilian wandering spider)	√	-	[55]
		*P. reidyi* (Para, Brazil)	√	-	[55]
		*P. reidyi* (Roraima, Brazil)	√	-	[55]
Hexathelidae	*Hadronyche*	*H. cerberea* (male and female) (Australian funnel-web spider)	√	-	[40]
		*H. infensa* (Australian funnel-web spider)	√	√	[40,56]
	*Illawarra*	*I. wisharti* (male) (Australian funnel-web spider)	√	-	[40]
Lycosidae	*Lycosa*	*L. singoriensis* (Chinese wolf spider)	-	√	[57]
		*L. vittata* (wolf spider)	-	√	[58]
Pisauridae	*Dolomedes*	*D. fimbriatus* (raft spider)	-	√	[59]
		*D. mizhoanus* (fishing spider)	-	√	[60,61]
		*D. sulfurous* (fishing spider)	-	√	[61]
Plectreuridae	*Plectreurys*	*P. tristis* (primitive hunting spiders)	√	√	[62]
Sicariidae	*Loxosceles*	*L. gaucho* (Brown spider)	√	-	[63]
		*L. intermedia* (brown recluse spider)	√	√	[64,65]
		*L. laeta* (Chilean recluse spider)	-	√	[66]
Scytodidae	*Scytodes*	*S. thoracica* (spitting spider)	√	√	[67]
Theraphosidae	*Acanthoscurria*	*A. paulensis* (Brazilian giant black tarantula)	√	-	[68]
	*Chilobrachys*	*C. jingzhao* (Chinese earth tiger tarantula)	√	√	[69,70]
	*Citharischius*	*C. crawshayi* (king baboon spider)	√	√	[71]
	*Grammostola*	*G. iheringi* (Argentinean black tarantula)	√	-	[72]
	*Haplopelma*	*H. hainanum* (Chinese bird spider)	√	√	[73,74,75]
		*H. schmidti* (Chinese bird spider)	√	√	[76,77,78,79,80]
Theridiidae	*Latrodectus*	*L. geometricus* (brown widow spider)	-	√	[81]
		*L. hesperus* (Western black widow spider)	√	√	[81,82]
		*L. tredecimguttatus* (Mediterranean black widow)	√	√	[83,84,85]
	*Steatoda*	*S. grossa* (cupboard spider)	-	√	[81]

**Table 4:** Proteomics and transcriptomics studies performed on scorpion venoms.

Family	Genus	Species	Prot.	Tran.	Reference
Buthidae	*Androctonus*	*A. bicolor* (black fat-tailed scorpion)	√	√	[86]
		*A. mauretanicus mauretanicus* (fat-tailed scorpion)	√	-	[87]
	*Buthacus*	*B. macrocentrus* (Turkish scorpion)	√	-	[88]
	*Buthus*	*B martensi* (Chinese Scorpion)	√	-	[89]
	*Centruroides*	*C. tecomanus*	√	√	[90]
	*Hottentotta*	*H. conspersus* (Sesriem scorpion)	-	√	[91]
		*H. judaicus* (black scorpion)	-	√	[92]
	*Isometrus*	*I. maculatus* (lesser brown scorpion)	-	√	[93]
	*Leiurus*	*L. quinquestriatus hebraeus* (yellow scorpion)	√	-	[94]
	*Leiurus*	*L. quinquestriatus quinquestriatus* (deathstalker scorpion)	√	-	[94]
	*Lychas*	*L. mucronatus* (Chinese Swimming Scorpion)	-	√	[93]
	*Mesobuthus*	*M. eupeus* (lesser Asian scorpion)	√	√	[95]
	*Rhoplaurus*	*R. junceus* (Caribbean blue scorpion)	√	-	[96,97,98]
	*Tityus*	*T. bahiensis* (Brazilian scorpion)	√	√	[94,99]
		*T. cambridgei* (Amazonian scorpion)	√	-	[100]
		*T. costatus* (Brazilian scorpion)	√	-	[101]
		*T. discrepans* (Venezuelan scorpion)	√	-	[102]
		*T. pachyurus* (Colombian scorpion)	√	-	[103]
		*T. serrulatus* (Brazilian scorpion)	√	√	[104,105,106,107,108,109,110,111,112]
		*T. stigmurus* (Brazilian scorpion)	√	-	[94,113,114]
Caraboctonidae	*Hadrurus*	*H. gertschi*	-	√	[115]
Chaerilidae	*Chaerilus*	*C. tricostatus*	-	√	[116]
		*C. tryznai*	-	√	[116]
Euscorpiidae	*Scorpiops*	*S. jendeki*	-	√	[117]
		*S. margerisonae*	-	√	[93]
Hemiscorpiidae	*Hemiscorpius*	*H. lepturus*	√	-	[118]
		*H. persicus*	√	-	[118]
	*Opisthacanthus*	*O. cayaporum* (Brazilian scorpion)	√	-	[119]
		*O. elatus*	√	-	[120]
Scorpionidae	*Heterometrus*	*H. longimanus* (black emperor scorpion)	√	-	[121]
		*H. petersii* (Asian forest scorpion)	√	√	[122]
	*Pandinus*	*P. cavimanus* (Tanzanian red clawed scorpion)	-	√	[123]
	*Scorpio*	*S. maurus palmatus* (chactoid scorpion)	√	√	[124]
	*Urodacus*	*U. yaschenkoi* (inland robust scorpion)	√	√	[125,126,127]

**Table 5:** Reported small molecules with inhibitory effect against scorpion and spider toxins.

Antitoxin	Chemical class	Molecular Formula	Structure	MW (Da)	Target	Target	Ref.
					Species	Toxin Family	
**Heparin**	Sulfated glyco-aminglycan	C_12_H_19_NO_20_S_3_	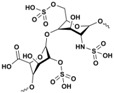	12–15 kDa	*Buthus martensi* (Chinese red scorpion), *Palamneus gravimanus* (Indian black scorpion), *Heterometrus fulvipes* (giant forrest scorpion)	Hyaluronidase	[129,130,131]
**Aristolochic acid**	Alkaloid	C_17_H_11_NO_7_	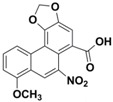	341.27	*Tityus serrulatus* (Brazilian yellow scorpion)	Hyaluronidase PLA_2_	[132]
**EDTA**	Acyclic	C_10_H_16_N_2_O_8_	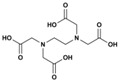	292.24	*Buthus martensi* (Chinese red scorpion), *Heterometrus fulvipes* (giant forrest scorpion), *Isonietrus vittatus* (scorpion), *Hemiscorpius lepturus* (scorpion), *Tityus serrulatus* (Brazilian yellow scorpion) *Loxosceles intermedia* (brown spider), *Hippasa partita* (spider), *Parawixia bistriata* (spider)	Metalloprotease Hyaluronidase PLA_2_	[129,131,133,134,135,136,137,138,139]
**1,10-phenanthroline**	Heterocyclic, 3-ring	C_12_H_8_N_2_	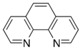	180.21	*Hemiscorpius lepturus* (scorpion) *Loxosceles intermedia* (brown spider), *Hippasa partita* (spider)	Metalloprotease Hyaluronidase PLA_2_	[133,134,138]

**Table 6:** Reported work on murine monoclonal antibodies and antibody fragments against spider toxins.

Name	Target	Type	Author	Year	Ref.
LiMAb(7)	*Loxosceles* *intermedia* (recluse spider).	mAb	Alvarenga et al.	2003	[149]
FM1	Alpha-latrotoxin from *Latrodectus* sp. (black widow spiders).	Fab	Bugli et al.	2008	[150]
LiD1mAb16	Sphingomyelinases D from *Loxosceles intermedia*, *L. laeta* and *L. gaucho* (brown or recluse spiders).	mAb	Dias-Lopes et al.	2014	[151]

**Table 7:** Reported work on murine monoclonal antibodies and antibody fragments against scorpion toxins.

Name	Target	Type	Author	Year	Ref.
mAb 4C1	Aah I from *Androctonus australis* (fat-tailed scorpion)	mAb	Bahraoui et al.	1988	[146]
mAb BCF2	Cn2 from *Centruroides noxius* (Mexican bark scorpion)	mAb	Zamudio et al.	1992	[152,153]
			Licea et al.	1996	
mAb 9C2	Aah II from *Androctonus australis* (fat-tailed scorpion)	mAb	Clot-Faybesse et al.	1999	[148]
scFv 4C1	Aah I from *Androctonus australis* (fat-tailed scorpion)	scFv	Mousli et al.	1999	[154]
mAbs BmK AS-1	BmK AS-1 from *Buthus martensii* karsch (Chinese scorpion)	mAb	Jia et al.	2000	[155]
scFv 9C2	Aah II from *Androctonus australis* (fat-tailed scorpion)	scFv	Devaux et al.	2001	[156]
chFab-BCF2	Cn2 from *Centruroides noxius* (Mexican bark scorpion)	chFab	Selisko et al.	2004	[157]
rFab 9C2	Aah I from *Androctonus australis* (fat-tailed scorpion)	rFab	Aubrey et al.	2004	[158]
Triple mutant (G5 + B7)	Cn2 from *Centruroides noxius* hoffmann (Mexican bark scorpion)	scFvs	Juárez-González et al.	2005	[159]
mAbTs1	TsVII, TsIV and TsNTxP from *Tityus serrulatus* (brazilian yellow scorpion)	mAb	Alvarenga et al.	2005	[160]
T94H6	Aah I and Aah II from *Androctonus australis* (fat-tailed scorpion)	Tandem-scFv	Juste et al.	2007	[161]

**Table 8:** Reported work on non-murine recombinant antibodies and antibody fragments against scorpion toxins.

Name	Target	Type	Author	Year	Ref.
scFv 3F, scFv 6009F	Cn2 from *Centruroides noxius* *hoffmann* (Mexican bark scorpion)	Human scFv	Riaño-Umbarila et al.	2005	[162]
NbAahI’22	AahI’ from *Androctonus australis* (Sahara scorpion)	Camelid Nb	Hmila et al.	2008	[163]
NbAahII10	AahII from *Androctonus australis hector* (Sahara scorpion)	Camelid Nb	Abderrazek et al.	2009	[164]
NbAah’F12	AahI from *Androctonus australis* (Sahara scorpion)	Camelid Nb	Hmila et al	2010	[165]
scFv 9004G	Cn2 from *Centruroides noxius* hoffmann and Css2 from *Centruroides suffusus suffusus* (Mexican bark scorpions)	Human scFv	Riaño-Umbarila et al.	2011	[166]
scFv LR		-	-	-	
scFv 15e	Ts1 or gamma-toxin from *Tityus serrulatus* (Brazilian yellow scorpion)	Human scFv	Amaro et al.	2011	[167]
NbF12-10	AahI and AahII from *Androctonus australis* (Sahara scorpion)	Bispecific Nb	Hmila et al.	2012	[168]
Db 9C2 + Db4 C1	AahI and AahII from *Androctonus australis* (Sahara scorpion)	Diabody mixture	Di Tommaso et al.	2012	[169]
Diabody D4, scFv LER	Cn2 from *Centruroides noxius* *hoffmann* (Mexican bark scorpion)	Diabody	Rodríguez-Rodríguez et al.	2012	[170]
Serrumab	Ts1 and Ts2 from *Tityus serrulatus* (Brazilian yellow scorpion)	Human scFv	Pucca et al.	2012, 2014	[171,172]
scFv C1	Cn2 from *Centruroides noxius* hoffmann (Mexican bark scorpion)	Human scFv	Riaño-Umbarila et al.	2013	[173]
scFv 202F	CII1 from *Centruroides limpidus limpidus* (Mexican bark scorpion)	-	-	-	
scFv RU1	Cn2 from *Centruroides noxius* hoffmann (Mexican bark scorpion), and CII1 from *Centruroides limpidus limpidus* (Mexican bark scorpion)	Human scFv	Riaño-Umbarila et al.	2016	[174]
scFv ER-5	Cn2 from *Centruroides noxius* Hoffmann (Mexican bark scorpion), CII1 from *Centruroides limpidus limpidus* (Mexican bark scorpion), and Css2 from *Centruroides suffuses suffuses* (Mexican bark scorpion)	Human scFv	Rodríguez-Rodrígues et al.	2016	[45]

**Table 9:** Reported work on next generation immunization strategies for spider antivenoms.

Immunization Strategy	Target	Author	Year	Ref.
Recombinant toxin	A dermonecrotic toxin from *Loxosceles* *intermedia* (recluse spider)	Araujo et al.	2003	[186]
Synthetic epitope	A dermonecrotic toxin from *Loxosceles* *intermedia* (recluse spider)	Felicori et al.	2009	[187]
Synthetic toxin	Robustoxin from *Atrax robustus* (Sydney funnel-web spider)	Comis et al.	2009	[188]
Recombinant toxin	A dermonecrotic toxin from *Loxosceles* *intermedia* (recluse spider)	Mendes et al.	2013	[189]

**Table 10:** Reported work on next generation immunization strategies for scorpion antivenoms.

Immunization Strategy	Target	Author	Year	Ref.
Synthetic epitope	AaH2 from *Androctonus australis* (Sahara scorpion)	Bahroui et al.	1986	[163]
Synthetic epitope	Cn2 from *Centruroides noxius* (Mexican bark scorpion)	Calderon-Aranda et al.	1995	[191]
Recombinant toxin	BotXIV from *Buthus occitanus tunetanus* (common European scorpion)	Bouhaouala-Zahar et al.	1996	[192]
Synthetic epitope	AaH2 from *Androctonus australis hector* (Sahara scorpion)	Devaux et al.	1997	[193]
Synthetic epitope	Cn2 from *Centruroides noxius* Hoffmann (Mexican bark scorpion)	Calderon-Aranda et al.	1999	[194]
Recombinant toxin	TsNTxP from *Tityus serrulatus* (Brazilian yellow scorpion)	Guatimosim et al.	2000	[195]
Recombinant toxin	AaH1, AaH2 and AaH3 from *Androctonus australis* (Sahara scorpion)	Legros et al.	2001	[196]
Recombinant toxin	Bot III from *Buthus occitanus tunetanus* (common European scorpion)	Benkhadir et al.	2001	[197]
Synthetic epitope	TsNTxP and TsIV from *Tityus serrulatus* (Brazilian yellow scorpion)	Alvarenga et al.	2002	[198]
Recombinant toxin	Cn5 from *Centruroides noxius* Hoffmann (Mexican bark scorpion)	Garcia et al.	2003	[199]
Synthetic epitope	Birtoxin from *Parabuthus transvaalicus* (South African fat-tail Scorpion)	Inceoglu et al.	2006	[200]
Recombinant toxin	Ts1 from *Tityus serrulatus* (Brazilian yellow scorpion)	Mendes et al.	2008	[201]
Recombinant toxin	PG8 from *Parabuthus granulatus* (granulated thick-tailed scorpion)	García-Gómez et al.	2009	[202]
Recombinant toxin	Css2 from *Centruroides suffusus suffusus* (Mexican bark scorpion)	Hernández-Salgado et al.	2009	[203]
Synthetic epitope	TsNTxP from *Tityus serrulatus* (Brazilian yellow scorpion)	Duarte et al.	2010	[204]
